# Morphological variations of the calcaneal tendon: clinical significance

**DOI:** 10.1186/s13018-023-03748-y

**Published:** 2023-04-04

**Authors:** Nicol Zielinska, Robert F. LaPrade, Łukasz Olewnik

**Affiliations:** 1 Department of Anatomical Dissection and Donation, Medical University of Lodz, Lodz, Poland; 2 Twin Cities Orthopedics, Edina, MN USA

**Keywords:** Accessory soleus muscle, Achilles tendinopathy, Calcaneal tendon, Adults, Calcaneal tendon, Embryology, Fetuses, Gastrocnemius muscle, Soleus muscle, Review

## Abstract

The calcaneal tendon, the largest and strongest in the human body, is created by the common junction of tendons of the gastrocnemius and soleus muscles. It is not a homogenous structure, being represented by layers in various arrangements. Morphological variability can be seen in the connection between the aponeurosis of the gastrocnemius muscle and the soleus muscle. Some types of plantaris tendon can be associated with a higher possibility of Achilles tendinopathy. Moreover, the presence of accessory structures, such as an accessory soleus muscle or additional gastrocnemius muscle heads may result in symptomatic pathologies. The main aim of this review is to summarize the current state of knowledge regarding the calcaneal tendon. Another aim is to present morphological variations of the calcaneal tendon and their clinical significance. Such information may be useful for clinicians, especially orthopedists, and surgeons. This review also provides an overview of embryological development and morphological variation among fetuses. Materials and methods: review was conducted according to PRISMA guidelines. An electronic search was conducted in five databases. Top quality tools were used to assess the quality of evidence in the studies reviewed. Research papers that made up the database of this review were analyzed, selected and assessed by two independently working researchers.

## Introduction

The calcaneal tendon (CT) is the largest and strongest tendon in the human body. It is created by a common junction of tendons of the gastrocnemius muscle (GM) and the soleus muscles (SM). Its insertion is located on the calcaneal tuberosity.

The CT is supplied by vessels from the posterior tibial artery, which are located in the paratenon. Blood is provided to the distal portion of the CT by the vessels from an arterial periosteal plexus on the posterior aspect of the calcaneus [1]. However, the middle portion of the CT has the greatest clinical interest, as the blood flow and supply is relatively lower in this part, resulting in more frequent tendinopathy and longer recoalescence [1].

The CT mainly receives sensory innervation by the sural nerve, but some small branches from the tibial nerve are also involved [2]. An important function is the Achilles deep tendon reflex: healthy people demonstrate plantar flexion of the foot in response to a blunt impact on the CT, which is used as a lower extremity neurological exam [3].

The CT is not a homogenous structure and is formed of various layers, which may be morphologically variable [1]. The CT may also differ in shape [4]. In some cases, the plantaris tendon (PT) may be fused with the CT, and inserts to the calcaneus as the CT [5]. However, this is only observed in rare cases, because the distal part of the plantaris muscle (PM) usually courses and inserts anteromedially or medially to this structure [5].

The main purpose of this review is to summarize the information about the CT available in current literature. Another aim is to present morphological variations of the CT, and discuss its clinical significance. Such information may be useful for clinicians, especially for orthopedists, and surgeons.

## Embryology

Differentiation of the common flexor muscular mass into the muscle rudiments may be observed in 11 mm embryos. After that, in most 14 mm embryos, two separate muscular groups can be seen. The first is a superficial group (proximolateral) consisting of the GM, SM, and PM and the second, the deep one (located medially), contains the flexor hallucis longus, flexor digitorum longus, popliteus, and tibialis posterior muscles. Connections can be seen between the GM group and blastemal of the calcaneus, and between the two long flexor muscles with the flat aponeurotic, from which there is an extension of tendons to the blastema of digits. Next, the GM-SM group spreads from the lateral to the medial side of the leg to attach to the tibia. In the second half of the second month of embryonic growth, both heads of the GM develop, particularly the lateral head. The last stage in the embryological development of the CT is the splitting off of the PM from the lateral head of the GM [6].

## Morphological variations of the calcaneal tendon and their clinical significance

### Layers of the calcaneal tendon

The CT is composed of several layers. A microscopic study by Szaro et al. [1] found that four groups of fibers take part in the creation of the CT. The posterior and lateral layers comprise fibers from the medial head of the GM. However, the anterior layer is created by fibers from the lateral head. Finally, the anteromedial part comprises fibers from the SM [1]. The CT elements are arranged spirally, and so divide the CT into three equal parts (around 5 cm in length): proximal, middle, and distal. The proximal part is represented by fibers arranged parallel to each other. The mid-part demonstrates some rotation, and the distal part full rotation [1]. At that level the fibers from the medial head of the GM are localized posteriorly (superficial part), those from the lateral head of the GM are localized anteriorly (deep part), and those from the SM form the central and medial parts. And as a result of such twisting, margins of the CT are represented by different fibers. The SM may also be involved in creating the posterior margin. The medial margin is represented only by the fibers from the SM. The fibers from the medial head of the GM constitute the lateral margin of the CT [1].

The distal part of the CT may be represented by morphological variations. Szaro et al. [1] indicated that in 90% of studied specimens, the major part of the posterior layer of the CT was created by GM fibers. Only in 10% was the major part was mainly represented by SM fibers. It was also reported that in 57.5% of studied lower limbs, the anterior layer was mainly represented by fibers from the lateral head of the GM. In 27.5% the involvement of SM and GM fibers (from the lateral head) was equal. Only in 15% of studied specimens was had greater involvement of SM than GM fibers [1].

Cummins et al. [7] classified the arrangement of the CT into three types, based on the percentage commitment of both heads of the GM and the SM. Type I was 2/3 SM fibers, Type II comprised equal amounts of GM and SM, and Type III was 2/3 GM [7].

Knowledge about CT layers may be useful during the diagnostic process of partial Achilles ruptures [8], which in most cases is related to inter alia soccer, track and field, basketball or running. Such pathology may be a result of acute injury or repeated microinjuries. The most vulnerable part is that which is the most loaded. A diagnosis can be complicated because the symptoms are similar to those in other CT pathologies. Most patients complain only about constant pain or swelling.

The most accurate and correct diagnosis can be made using imaging techniques like MRI or US. The precise visualization of the internal structure of the CT and an indication the exact level and range of involved fibers is possible, and is used by clinicians to monitor the treatment process [9]. The gold standard for treating partial ruptures of the CT is an operative reconstruction, which depends on end-to-end suturing or additional autogenic tissue autograft (in most cases, the PT is used) [10].

CT partial injuries were classified based on histologic and anatomic characteristics. Regarding the former, Type A concerned acute injury (early reparative process) and Type B concerned chronic injury (no reversible degenerative changes) [11]. Regarding the latter, Type I was represented by an injury within one bundle, Type II within two bundles, and Type III by injury within three bundles, the letter being complete rupture of the CT. These injuries may involve fibers originating from the SM (signed in this classification as ‘S’), from the medial head of the GM (as ‘GM’), or from the lateral head of the GM (as ‘GL’).

Thus, a chronic partial injury of fibers originating from the SM and lateral head of the GM may be classified as B-II-S-GL.

### Morphological variability of connection between aponeurosis of the gastrocnemius muscle and the soleus muscle

The CT is formed by fibers from both the GM and the SM. However, the place of fusion of these structures may be morphologically variable. For example, the GM can insert directly onto the tendinous superficial surface of the SM. However, in most cases, the GM is inserted to the SM by its aponeurosis. The length of this aponeurosis may also vary.

Blitz et al. [12] classify short aponeurosis as those measuring 10 mm or less (separately for medial and lateral side) and long ones as greater than 10 mm. When the GM heads were not represented by aponeurosis, the attachment to the SM aponeurosis was classified as direct. The most common type (53%), was a long aponeurosis, both medially and laterally, while 9% were represented by a short aponeurosis (medial) and long aponeurosis (lateral). A direct attachment to the SM aponeurosis was observed in 38%, and a direct attachment to only the GM medial head in 23%, and lateral fibers formed short (6%) or long (17%) aponeurosis. In 11% of the studied lower limbs, a direct attachment was found for both GM heads. In turn, 4% demonstrated a direct attachment of the lateral head only, and a short aponeurosis located medially. No examples were found of the co-occurrence of a long aponeurosis in the medial part and a short lateral aponeurosis or a direct lateral attachment, nor of the co-occurrence of a short medial and lateral aponeurosis.

Knowledge about such variations seems to be necessary during GM recession (release or lengthening of the GM aponeurosis). Only an aponeurosis which is not connected with the muscular part may be used during surgical lengthening; therefore, information regarding the aponeurotic attachment is valuable.

In most cases it is easy to separate a long GM aponeurosis from a deeply-located SM aponeurosis. When the GM aponeurosis is short, it seems to be more difficult. Moreover, when the direct attachment of the GM to the SM occurs, it is impossible to complete surgery without surgically detaching the GM from its direct attachment, or without cutting the SM aponeurosis, resulting in an increased risk of side effects and a longer recovery.

Blitz et al. [13] report that a medial short aponeurosis is often accompanied by a long lateral GM aponeurosis, and in most cases there should not be any problems during surgery, assuming that the lateral part is not represented by direct attachment of the lateral head of the GM to the SM. This is important when using a medial approach to recess the GM. The narrower part of the aponeurosis is easier to find. The most common subtype of direct attachment was a single direct attachment of the medial head of the GM; this was correlated with an average length of the lateral aponeurosis (18 mm). Hence, when the medial GM head is surgically released in the first stage of the operation, the next stage, releasing the lateral GM aponeurosis, should be non-problematic. However, the features of the lateral aponeurosis should also be taken into consideration, because in three cases of all represented by the direct medial head attachment, the lateral aponeurosis was assessed as long, which may complicate surgery.

Another type was represented by direct attachment of both, the medial and lateral heads of the GM, and such arrangements seems to be the most problematic in recession the GM.

In some cases, only the lateral head of the GM was directly attached to the SM, but no correlation was found with the medial aponeurosis: one case was classified as long, and another as short.

An interesting conclusion from this study is that the medial side of the GM is more restricted than the lateral one. So, there is a higher possibility that the medial part (in relation to the lateral part) will be represented by the direct attachment, or short aponeurosis. When the medial and lateral aponeurosis are classified as a long type, there is also a higher possibility that the medial one will be shorter than the lateral. Taking everything into consideration, the medial or posterior approach during GM recession seems to be an easier technique (the medial side with shorter or absent aponeurosis will be better to visualize) [12].

## Morphological variations of the calcaneal tendon in human fetuses

Szaro et al. [14] report CT rotation in all tested human fetus specimens, and a similar arrangement of layers to adults: fibers from the GM lateral head form the anterior layer of the CT, those from the medial head form the posterior layer of the CT, and those from the SM form the anteromedial part of the CT.

The morphology of the posterior (superficial) layer of the CT was also assessed, and two types were distinguished. In most cases (94.4%) this layer was only formed by fibers from the medial head of the GM. In the remaining 5.6%, it was also formed by fibers from the SM, but with GM medial head fibers dominating [14].

The medial part of the CT demonstrated four patterns of formation. The most common comprised only SM fibers (68.1%). In 27.8% of studied specimens, SM fibers dominated over the GM. In 4.2%, co-domination or GM domination was observed [14].

In most cases, the lateral part of the CT, is formed only by fibers from the GM medial head (79.2%). However, in 15.3% the GM fibers predominate over GL. Cases with only GL fibers or with GL > GM are very rare [14].

Edama et al. [15] distinguished three types of CT deep layer among fetuses. Type I was represented by fibers running only from the SM. This type occurred in 13% of studied cases, and it was classified as a ‘least twist’. The most common type was Type II (77%), represented by the deep layer of the CT formed by fibers from both the GL and SM; this was classified as a ‘moderate twist’. The final type was formed only by GL fibers; it occurred in 10% of the studied specimens, and was classified as ‘extreme twist’ [15].

The above division for the layers and twisting of the CT is observed in fetuses in their second trimester of pregnancy [14, 15].

## Morphological variations of plantaris tendon insertion

The course of the PT and CT is close, and their relationship to each other may be variable. This is the reason why morphological variations of the PT course and place of insertion, or its connection with the CT may be clinically significant, for example, in Achilles tendinopathy. Knowledge about various types of the distal part of the PT is important for clinicians, especially for orthopedists, surgeons, or physiotherapists.

Olewnik et al. [16, 17] carried out a study which assessed the insertion of the PT and its arrangement in relation to the CT. Six types of it were distinguished. The first one was a standard type (44%), in which the PT was distally attached as a wide, fan-shaped tendon to the calcaneal tuberosity, medially to the CT. Type II (22.4%), was represented by a distal connection between the located medially PT and CT, attached to the calcaneal tuberosity. Type III (6.9%) was characterized by a distinct PT attached to the calcaneus, anteriorly to the CT. In Type IV (3.4%), the PT coursed also anteriorly to the CT, but it was an interesting type: there was no distal attachment placed on the calcaneus, but the PT was inserted to the deep crural fascia. In turn, Type V (18.1%) was characterized by a wide distal attachment encircling the medial and posterior part of the CT. Finally, Type VI (5.2%) demonstrated a PT inserted at a point near to the tarsal canal flexor retinaculum of the leg [17].

Also, the course of the PT in relation to the CT was assessed, and Olewnik et al. [17] distinguished two types of it. Type I (84.5%) was represented by a PT initially located between the GM and SM, after which, it was located on the medial side of the CT. In Type II (15.5%), the PT was also initially located between the GM and SM, but then ran directly anterior to the CT.

Olewnik et al. [16] also carried out a similar study a year earlier, but there were no Type VI in the studied population (Types I-V were the same as these mentioned above). In this study, the frequency was: Type I—44%, Type II—18%, Type III—8%, Type IV—4%, and Type V—22% [16].

Another study was carried out among fetuses [18]. Eight types of PT insertion were distinguished. The first six types were the same as those described by Olewnik et al. [17]. The first new type (Type VII) was represented by a PT highly inserted to the CT (10.9%). In one case, the PT was divided into two structures before attachment to the CT. Type VIII (2.2%) was characterized by a short tendon and was distally attached to the SM.

The relationship between the PT and CT was also studied by Sterkenburg et al. [19], however, nine different types of insertion of the PT were distinguished. The PT was separated from the CT in 89.7% of cases and joined in 10.3%. Moreover, in three specimens, the PT and CT were connected to each other by a retinaculum-like structure (32–88 mm proximally to place of distal attachment). In another three cases, the PT was attached to the CT at the level of its mid-portion (20–65 mm proximally to place of distal attachment). Other types in which a close connection was observed between PT and CT were inserted to the deep fascia (two cases), anteromedial side of the CT into the calcaneal bone (two cases), and onto the anterior side of the CT into the calcaneus (one case) [19].

In a study by Nayak et al. [20], 26.92% of the studied population demonstrated a connection between the PT and the CT at various levels. In other cases, the PT was inserted separately to the calcaneus, or to the flexor retinaculum of the foot.

Cummins and Anson [7] also examined the place of insertion of the PT, and its relationship to the calcaneal tendon. In most cases, the PT was represented as a distinct distal attachment, medially (47%) or anteriorly (36.5%). In some cases, a connection was noted between the PT and CT—a broad insertion investing the posterior and medial part on the adjacent CT (12.5%), or an insertion into the medial border of the CT, 1–16 cm proximally to the level where the CT is attached to the calcaneus.

Gonera et al. [21] described a case in which the insertion of the PT was represented by four distinct tendons; however, none of them were attached to the CT. The first inserted to the tarsal canal flexor retinaculum (located medially to the CT), the second was divided into two smaller structures inserted to the calcaneal tuberosity posteriorly to the CT insertion: the first attached medially, and the second laterally. The third band was located anteriorly to the CT, and inserted also to the calcaneus [21].

Kurtys et al. [22] found a PT that was initially located between the SM and medial head of the GM, and then ran medially to the CT (as in most cases). However, its next part was divided into two bands (A and B), and both were divided into two tendinous insertions. Band A was divided into two smaller tendons: the first was a fan-shaped structure attached to the superomedial part of the calcaneal tuberosity and the second was a straight, plain band attached to the medial part of the calcaneus body. Band B was represented by two smaller structures: the first was distally attached to the superior surface of the calcaneal bone, anteriorly to the CT (near to its medial ridge), and the second inserted to the medial part of the calcaneus body. Another additional tendon was observed, which was a connection between band A and the anteromedial surface of the CT, located perpendicular to them [22].

It is worth mentioning that the PM may be absent, but at various frequencies. Olewnik et al. [16] found the PM to be absent in 4%, while a second study [17] found this to be 10.8%. In turn, Harvey et al. [23] noticed that the PM was absent in 19%. Simpson et al. [24] found this muscle to be absent between 7 and 20% of cases. Nayak et al. report absence in 7.69% of adults and 22% of fetuses [25].

Interestingly, Meyer et al. [26] carried out a study in which compared the results of cadaveric anatomical dissection, and ultrasonography examinations on patients. It turned out, that the PT was present in all cadavers, but absent in 4.4% of patients; however, the sample of cadavers was significantly smaller (six cadavers) than patients (335 patients).

## Clinical significance of plantaris muscle morphological variations

Although the PT is not usually involved in forming the CT, its proximity may increase the risk of occurrence of Achilles tendinopathy, especially in its middle portion. Olewnik et al. [17] distinguished specific morphological types of the PT whose presence may be associated a higher risk.

Type II PT [17], represented by a distal connection between the medially-located PT and CT, attached to the calcaneal tuberosity, may be strongly connected with mid-portion Achilles tendinopathy. A higher risk of tendinopathy may be associated with a specific course of a Type II PT from between the GM and SM to the medial side of the CT.

In addition, Type I, characterized by a wide, fan-shaped tendon inserted to the calcaneal tuberosity, medially to the CT, may increase the risk of tendinopathy, as may Type V, characterized by a wide distal attachment encircling the medial and posterior part of the CT, and Type VI, by an insertion at a point near to the tarsal canal flexor retinaculum of the leg [17].

It has been proposed [17] that an absent PM may not be correlated with a higher risk of Achilles tendinopathy. However, more clinical studies are required to improve it.

In one study, patients with Achilles tendinopathy demonstrated a thickened PT. It was located very close to the CT, so during surgery, not only the ventromedial CT was debrided, but also the PT was excised. Patient satisfaction was assessed 6 months after the operation: 87.5% of patients were satisfied.

The prevalence of the correlation between the Achilles tendinopathy and PT depends on the study and examined population. This can range from 79.5% of studied population [27] to 67.2% [28].

The PT has also a positive clinical significance associated with the CT. It is commonly used in surgical treatment of CT rupture. It is a common belief that the PT with its bony insertion (without connection with the CT) is a reliable source for such operation. However, before this procedure, US and MRI should be performed to highlight additional structures associated with the PT and improve the surgical treatment plan. For example, cases in which the PT is represented by four separate tendinous insertions [21, 22], may be problematic for clinicians, and prolong the operative time. On the other hand, these additional tendinous structures may be used as autograft material without using the main PT tendon, thus decreasing possible side effects, and accelerating recovery.

US and MRI can also be used to confirm the absence of the PT, and their use can hence avoid unnecessary procedures and tissue damage.

## Morphological variations of the soleus muscle

The SM has a number of morphological variations in the muscle fibers. Joshi et al. [29] divided them into bipennate, unipennate, and double pennate, with the first being the most common (86%).

Olewnik et al. [30] report a bipennate and unipennate SM, but additionally distinguished the multipennate type (including double pennate) and non-pennate (which has not been described previously), and created a new classification to accommodate them. Type I (43.8%) was represented by a bipennate muscle, whose fascicles were attached to both sides of the central tendon creating the CT with the tendon of the GM. Type II (15%) was characterized by a unipennate muscle, and its fascicles (resembling one half of a feather) run at an acute angle on one side of the central tendon, which takes part in creating the CT with the GM. Type III (5%) a ‘multipennate muscle’, was represented by fascicles attached to many tendons within the muscle; however, only one central tendon formed the CT with the GM. Finally, Type IV (36.3%) is a non-pennate type lack any central tendon in its muscular part; the SM took part in forming the CT [17].

Pedret et al. [31] distinguished different types of the SM on the basis of their muscular and connective dominance. The central tendon divides the SM into two muscle volumes: one to the medial border and another to the lateral border. One volume might be bigger than the other, or they may be the same size (symmetrical). If no central tendon was present, no dominance was assessed [31]. A symmetrical type, the central tendon is in the middle, and medial and lateral part are symmetrical. The next type was represented by medial muscle dominance, in which the medial part of the SM had greater muscle volume than the lateral one and the central tendon was located laterally. The next type was represented by non-muscular dominance, with a lack of any central tendon, or with the presence of several hypoplastic tendons. The next is characterized by medial connective dominance, with the medial aponeurosis being longer and thicker than the lateral one. The final type is similar, but with the lateral aponeurosis dominating [31].

The variability of the SM may also depend on presence of some additional structures, like the accessory soleus muscle of Cruveilhier [32]. This structure may originate from the soleal line of the tibia, or from the SM, or the proximal third of the fibula. In its distal part it courses anteriorly or antero-medially to the CT. The place of insertion may be variable. Hatzantonis et al. [33] distinguished three different types. The first one was attached to the calcaneus as a separate tendon located medially to the CT. The second type was represented by an insertion to the CT. The third type was a tendinous or fleshy attachment bifurcated and inserted onto the medial part of the calcaneal bone. It is worth mentioning that the accessory soleus muscle is located superficial to the flexor retinaculum [33].

Another variation is the tibiocalcaneus internus muscle (of Testut) [34]. It originates from the soleus line and medial crest of the tibia. Its insertion is located on the medial part of the calcaneus, anteriorly to the CT. The main thing that sets this muscle apart from the accessory SM, is that the tibiocalcaneus internus muscle is located deep to the flexor retinaculum, or within the tarsal tunnel (superficial to the neurovascular structures) [34].

Interestingly, the SM may be completely absent, and in such cases, the CT is formed only by the tendon of the GM. However, such absences are very rare [35].

## The clinical significance of morphological variations of the soleus muscle

SM domination and the presence, or absence, of the central tendon may be clinically important. It has been proposed that involvement of the connective tissue has a worse prognosis than the involvement of the muscular mass. Hence, it may be concluded that because it depends on involvement of connective structures, i.e. the SM central tendon and aponeurosis, muscular domination has no great influence on recovery time. So, an affected SM represented by non-muscular dominance, and a lack of central tendons (or hypoplastic tendon), is associated with better prognosis during recovery process, than one represented by a long central tendon.

In a study of SM tears, 54.4% were associated with the proximal part of the SM, and 45.6% with the distal one. The anterior aponeurosis was affected in 25.3%, and the posterior aponeurosis in 20.25%. In 19% the torn part was the medial intramuscular aponeurosis, and in 20.25% the lateral one. The central intramuscular tendon of the SM, which may force longer recovery, occurred in 15.2% [36]

In some cases, the accessory soleus muscle is solitary. It may be asymptomatic and detected occasionally during imaging examination. If this additional structure is symptomatic, the usual presentation is a soft mass which may be palpated in the posteromedial distal third of the leg. Sometimes, physical activity can cause an increase in its size. Some patients complain of pain and impaired physical function, especially during jumping and running. The most common pathology associated with an accessory soleus muscle is chronic compartment syndrome. In other cases, the neurovascular system may be compressed; posterior tibial artery compression may lead to inadequate blood supply. If the posterior tibial nerve is compressed, its function may be impaired [37]. In some cases, this muscle may be associated with clubfoot deformity among children. The diagnosis of this pathology is important, because of the possibility of hindfoot deformity. The anomalous muscle can also cause the tarsal tunnel syndrome, or Achilles tendinopathy.

## The morphological variations of the gastrocnemius muscle

Morphological variations of the GM may influence the presentation of the CT resulting in a higher occurrence of Achilles tendinopathy. Hence, clinicians, especially orthopedists and physiotherapists, should be aware of them [35].

In some cases, the GM is totally or partially absent [35]. Sometimes, the one head of the GM may be absent or not fully developed. In other cases, the muscular mass of the lateral head may be replaced with a tendinous band [35]. In most cases, both muscular heads are fused together before passing into common tendon; however, the medial and lateral heads of this muscle are sometimes separated from each other, then pass into tendinous also separated parts, and only merge together creating the CT close to the calcaneal tuberosity [35].

Both, the medial and lateral heads of the GM may be represented by two layers along their entire length. There is also the possibility that these heads may be replaced by fibrous bands [35].

In some cases, the GM and SM form the CT only near its attachment to the calcaneus, and in others, these muscles may have two distinct insertions, without the CT be created [35].

Some accessory structures associated with the GM may be present. For example, the gastrocnemius tertius, i.e. the third head of the GM. It originates from the popliteal surface of the femur, and more specifically from the linea aspera, lateral epicondyle, knee joint capsule, or middle part of the fibula. However, it can also originate from other muscles, like the semitendinosus muscle, or biceps femoris muscle (its long head). Studies have also described the gastrocnemius tertius arising from the crural fascia [13, 38, 39]. Its insertion is usually located on one of the GM heads, but sometimes is distally divided and attaches to both heads.

Yildirim et al. [40] described the co-existence of a bilateral gastrocnemius tertius muscle and unilateral accessory soleus muscle. Innervation for both muscles was from the tibial nerve. On the left side, the gastrocnemius tertius was two-bellied: the superficial head had its origin from an area located just above the PT, and the deep one was proximally attached to the PT. The superficial belly was inserted to the lateral head of the GM, while the deep belly was distally attached to the medial head of this muscle. The accessory soleus muscle had a proximal attachment on the posteromedial part of the tibia and soleal line of this bone. Its insertion was located on the medial area of the calcaneal bone, but there was no connection between the CT and this accessory structure. The right side differs from the left, because there was no occurrence of the accessory soleus muscle. Moreover, the gastrocnemius tertius was represented by one head proximally attached to the lateral condyle of the femur. It was distally attached to the medial head of the GM [40].

Ishii et al. [41] also describe a bilateral gastrocnemius tertius. In both sides, it originated laterally to the tibial nerve, and superiorly to the PM. Their tendons were fused with the investing fascia of the GM. The innervation was from the tibial nerve branches [41].

Another anomaly associated with the GM is the quadriceps gastrocnemius muscle. Ashaolu et al. [42] identified a four-headed GM not described previously in the literature. This morphological variation occurred bilaterally. The lateral head originated proximally to the lateral part of the lateral condyle of the femur and fibrous capsule. The interomedio-lateral head was represented only by a muscular part originating from the lateral half of popliteal surface of the femur. The interomedio-medial head was also represented only by a muscular part and its origin was located on the medial half of the popliteal surface of the femur. The medial head was proximally attached to the medial part of the medial condyle of the femur. All heads took part in forming the CT. Every head was separated from others by deep fascia. Innervation also occurred from distinct branches from the tibial nerve. An anomalous variation of the GM represented by four heads were named the quadriceps gastrocnemius muscle [42].

A study found the normal two-headed GM to be present in 93.33% of a studied population, with a quadriceps gastrocnemius muscle (four-headed GM) present in the remaining 6.66%. No three-head GMs were found. All heads in the four-headed GM were merged distally, and the CT created with the SM inserted onto the calcaneal bone [43].

However, another study found the two-headed (standard) GM to be present in only 35% of the studied population, with a three-headed GM in 13.3% cases. The most common type was the four-headed GM, present in 51.7%. In all cases, muscular parts of heads were distally connected and involved in creating the CT, inserted onto the calcaneal bone [42].

An interesting case was a six-headed GM [44]. The medial part of the muscle was represented by three heads originating from the medial femoral condyle and the adjoining part of the medial supracondylar line. The lateral part of the GM was also characterized by three heads; the most lateral one originated from the lateral femoral condyle, and the other two had their origin on the supracondylar line. All heads remained separate until forming the CT. Most importantly, the sural nerve passes between the medial and lateral part of the GM, and was piercing the GM at the level of formation of the CT [44].

## The clinical significance of morphological variations in gastrocnemius muscle

Additional structures may compress neurovascular structures, leading to clinical problems. The gastrocnemius tertius, one of the most common morphological variations associated with the GM, may cross nerves or vessels located in the popliteal region and lead to arterial and venous stasis, popliteal vascular entrapment syndrome, intermittent claudication, or impaired nerve function. The most problematic type of gastrocnemius tertius is the one that inserts to the medial head of the GM [35].

However, all additional heads of the GM may be associated with some pathologies. Popliteal artery compression may cause pain or cramping in the calf. Moreover, popliteal artery compression may result in cold feet (after activity) and paresthesia, represented by tingling, burning or numbness in the posterior region of the lower leg [45].

Sometimes, the popliteal vein may be also compressed, which leads to cramping in the lower leg (especially at night) and swelling in the calf area. Some patients complain of a heavy feeling in the affected leg. Other important symptoms include changes in skin color in this region, and possible deep vein thrombosis. As such, a Doppler examination may be advisable [35].

An occurrence of additional heads of the GM may also be associated with sural nerve compression. For example, a high possibility of compression has been associated with a six-headed GM and a sural nerve located between the medial and lateral parts of the muscle [45]. Patients with entrapment of the sural nerve usually complain of sensory alterations over the distribution area of the nerve: the postero-lateral side of the distal third of the leg, lateral part of the foot and fifth toe; however, this nerve may be variable in the dorsal part of the foot, so affected regions may differ between patients. Other symptoms are burning pain, dysesthesia, paresthesia or hypoesthesia, which may be aggravated after physical activity or during sleeping. Moreover, tenderness may also occur during palpation [46].

## Conclusions

The CT is not a homogenous structure, and is formed by fibers from both the GM and the SM and the place of fusion of these structures may be morphologically variable and clinically significance. Long GM’s aponeurosis predisposes to easy separation from a deeply-located SM’s aponeurosis during surgery. However, when the GM’s aponeurosis is short, it seems to be more difficult. Moreover, when the direct attachment of these two muscles occurs, it is impossible to complete surgery without surgically detaching the GM from its direct attachment, or without cutting the SM’s aponeurosis, resulting in an increased risk of side effects and a longer recovery. Other variations, for example, attachment of the PT to the CT or occurrence of additional structures may be also clinically significance and predispose to Achilles tendinopathy.

## Data Availability

The authors can be contacted for data requests (Łukasz Olewnik, PhD—email address: lukasz.olewnik@umed.lodz.pl).
